# Convalescent plasma for COVID-19: the risk of pulmonary embolism should not be underestimated!

**DOI:** 10.1186/s13054-020-03236-3

**Published:** 2020-08-28

**Authors:** F. Sanfilippo, V. La Rosa, F. Oliveri, M. Astuto

**Affiliations:** 1 Department of Anaesthesia and Intensive Care, A.O.U. “Policlinico-Vittorio Emanuele”, Catania, Italy; 2 School of Anaesthesia and Intensive Care, University Hospital “G. Rodolico”, University of Catania, Catania, Italy; 3 Department of General Surgery and Medical-Surgical Specialties, Section of Anesthesia and Intensive Care, University of Catania, Catania, Italy

Dear Editor,

We read with great interest the elegant editorial of Shankar-Hari et al. [1] regarding the need of randomized studies on the use of convalescent plasma in coronavirus disease (COVID-19) infection. The authors describe safety issues associated with the administration of convalescent plasma, reporting both non-specific (infection, circulatory overload, lung injury) and disease-specific (viral enhancement) side effects of plasma transfusions.

However, it is of utmost importance to highlight that COVID-19 represents a unique scenario where convalescent plasma may be harmful for another essential reason. Indeed, plasma is commonly administered in patients with coagulopathies and/or hemorrhage, as it contains pro-coagulant factors. Importantly, COVID-19 patients are at the other end of coagulative disorders, and it has been consistently shown they develop a pro-thrombotic disease with high-risk of pulmonary embolism [2]. Although laboratory pre-treatment of convalescent plasma with methods aiming at inactivating potential residual virus (such as methylene blue) may decrease also its pro-coagulant effects [3], the presence of even small quantities of coagulation factors may still trigger the coagulation cascade, potentially harming COVID-19 patients. Therefore, it is crucial to ascertain which (if) treatments during the preparation of convalescent plasma have been performed to reduce the amount of coagulation factors. Anyway, we believe that plasma transfusions should be administered thoughtfully in COVID-19 patients to avoid an unnecessary potentiation of their already existing pro-thrombotic state.

In this regard, we do not entirely share the recent enthusiasm generated by a preprint un-reviewed study where the convalescent plasma was administered to over 5000 patients with severe or life-threatening COVID-19 [4]. Indeed, these results should be taken cautiously as the reported low incidence of serious adverse effects refers to an extremely short time-frame of observation (4 h). This period is far too short to fully account for deteriorations due to the progression of the pro-thrombotic state which could potentially generate not only pulmonary embolism but also other systemic events such as cerebral ischemia. Interestingly, the efficacy of convalescent plasma for the treatment of severe influenza has been heavily questioned by a recent meta-analysis published in the journal [5].

To date, several randomized controlled studies are underway (as of the 30th of July on www.clinicaltrial.gov, we found nine studies completed, and 76 currently recruiting). These studies addressing the role of convalescent plasma in COVID-19 should specify the plasma pre-treatment to decrease the amount of coagulation factors and carefully evaluate the incidence of adverse thrombotic events in a timeframe much longer than 4 h.

## Data Availability

Not applicable
